# Pulse oximetry, racial bias and statistical bias

**DOI:** 10.1186/s13613-021-00974-7

**Published:** 2022-01-04

**Authors:** Martin J. Tobin, Amal Jubran

**Affiliations:** Division of Pulmonary and Critical Care Medicine, Hines Veterans Affairs Hospital and Loyola University of Chicago Stritch School of Medicine, Hines, IL 60141 USA

On November 21, 2021, the British Health Minister, Sajid Javid, published an article in the *Sunday Times* pointing out that pulse oximeters tend to be inaccurate in patients with dark skin pigmentation [1]. The Minister remarked that “technologies are created and developed by people, and so bias, however inadvertent, can be an issue here too.” The article attracted immediate and widespread attention, being covered by newspapers and television channels around the world. Mr. Javid expanded on his concerns during interviews with Andrew Maar on the *BBC* and Trevor Phillips on *Sky News*.

A few years after the introduction of pulse oximetry in the 1980s, we noticed that oximeters were less reliable in Black patients. In a 1990 article focusing on oxygenation in mechanically ventilated patients, we reported that pulse oximetry was almost 2½ times less accurate in Black patients [2].

Given that pulse oximetry operates by shining a light through the skin at two wavelengths—660 nm (red) and 940 nm (infrared)—and measuring the difference in light absorbance at the two wavelengths to estimate arterial oxygen saturation [3], we surmised that inaccuracy in Black patients is related to skin pigmentation. Supporting evidence is the increase in the difference in light absorbance between red and infrared wavelengths with use of black nail varnish, causing pulse oximeters to register falsely low saturations [4].

Estimates of oxygen saturation by pulse oximetry differ from true oxygen saturation in an arterial blood sample (the reference standard). The magnitude of the difference between non-invasive estimates and reference-standard measurements (in a group of patients) is quantified as mean and standard deviation, which statisticians dub “bias and precision.” In White patients, the mean and standard deviation of the difference between non-invasive estimates of oxygen saturation and the reference standard are each around 2%, whereas they both exceed 3% in Black patients [2]. In our study, a pulse oximeter target of 92% ensured a safe arterial oxygen tension (PaO_2_) greater than 60 mmHg in 91.7% of White patients, but in only 50% of our Black patients [2].

In the first rigorous description of happy hypoxia in COVID-19, a situation where patients experience life-threateningly low oxygen saturations without provoking dyspnoea, we discussed several contributors to the phenomenon including the greater unreliability of pulse oximetry in Black patients [5]. Managing patients with unreliable measurements of oxygenation is hazardous, partly because dangerously low saturations are missed, but also because low saturations are exaggerated and lead to unnecessary intubation [6]. A major contributor to patient mortality in COVID-19 is inappropriate intubation [7], and it is possible that unreliable measurements of the oxygen saturations have contributed to increased mortality reported in Black patients [8].

In our 1990 article, we deduced that pulse oximetry was less reliable in Black patients because calibration data were drawn largely from White subjects [2]. The algorithms employed within software of pulse oximeters are trade secrets and not open to scrutiny [3]. We recommended that manufacturers collect data in Black patients to develop better calibration algorithms. In the 31 years since we made this recommendation, we are not aware of any manufacturer attempting to incorporate adjusted algorithms into pulse oximeters. The inaccuracy of pulse oximetry in Black patients is one further example of how medical information generated in (and for) White people contributes to inferior clinical outcome in patients of colour [9].

Hopefully, the attention generated by Mr. Javid’s highlighting of a significant problem with pulse oximetry will rouse manufacturers into action. Mr. Javid is working with his US counterpart, Xavier Becerra, to introduce new international medical standards. In the meantime, it would help if authors reverted to the more mundane (and precise) terminology of mean and standard deviation and refrained from use of the dodgy terms “bias and precision”, which gives rise to the notion that inanimate objects are capable of racial prejudice.

## Data Availability

Not applicable.
